# Studies on the time course of apparent diffusion coefficient and signal intensities on T2- and diffusion-weighted MR Imaging in acute cerebral ischemic stroke

**DOI:** 10.4103/0971-6203.44479

**Published:** 2008

**Authors:** Ajai K. Srivastava, Gopesh Mehrotra, Satish K. Bhargava, Sunil Agarwal, Rajendra P. Tripathi

**Affiliations:** Department of Radiology, University College of Medical Sciences, (University of Delhi) and Associated GTB Hospital, Dilshad Garden, Delhi - 110 095, India; 1 Department of Medicine, University College of Medical Sciences, (University of Delhi) and Associated GTB Hospital, Dilshad Garden, Delhi - 110 095, India; 2 Institutes of Nuclear Medicine and Allied Sciences, Lucknow Road, Delhi, India

**Keywords:** Apparent diffusion coefficient, B-value, DWI, FLAIR, ischemic stroke, signal intensity

## Abstract

The time course of changes in apparent diffusion coefficient (ADC) and signal intensity on diffusion-weighted magnetic resonance imaging (DW MR) imaging in acute ischemic stroke is a very dynamic event. There is an initial reduction in ADCs with no change on T2-W imaging but signal intensity increase on T2-weighted takes place about 6–12 hours after onset of stroke. As necrosis begins to set in, there is a gradual reversal of ADC change, and around 3–10 days post-onset, ADC pseudonormalizes. Twenty-four patients of acute stroke underwent diffusion MR imaging in addition to conventional T1W, T2W, and Fluid Attenuated Inversion Recovery (FLAIR) sequence performed within 12 hours, at 30 days, and at 90 days. The mean signal intensity at b = 0 s/mm2 and at b = 1000 s/mm2 were significantly higher than control values for all time periods. The ratio of signal intensity at b = 0 (rSI b=0) significantly increased from 1.63 ± 0.20 in the acute stage to 2.19 ± 0.24 in the chronic stage (*P* < 0.001). The ratio of signal intensity on DWI (r SIDWI) decreased from 2.54 ± 0.46 to 1.54 ± 0.22. The mean ADC in the lesion was found to be 41% lower than the mean ADC in the contralateral hemisphere .Linear regression analysis between rADC and log hours showed that pseudonormalization occurred at 6.61 days (*P* < 0.001). We conclude that the above information could be useful in the management of very early stroke.

## Introduction

Strokes are commonly (approximately 70%) ischemic in origin.[1] The ability to identify ischemia in the first three hours is important for the choice of newer therapeutic interventions. Conventional MRI and CT can not be used to predict the presence and extent of ischemic damage in the hyperacute stage.[2–4] Moseley *et al.*[5] reported that diffusion-weighted (DW) MR images which provide image contrast dependent on microscopic, random, translational motion of water molecules known as (self-) diffusion, could be used to detect ischemic changes at a very early stage. In 1950, Hann described how molecular diffusion in the presence of a static gradient field, influences measured nuclear magnetic resonance signal intensities. Carr and Purcell et al. determined the diffusion coefficients of water with an improved experimental method, evaluating the spin echo (SE) after a 90° and an 180° radio frequency (RF) pulse. The echo signal amplitude of the n^th^ echo is given by:

(1) $$S\;(n\;\mathrm{TE})\;/\;S\;(O)\;=\;\exp[(-n\;\mathrm{TE})/T_{2}]$$

Where T_2_ = transverse relaxation time and TE = echo delay

Equation (1) becomes invalid in the presence of diffusion. The attenuation because of the random phase shift of spins (diffusion) depends on the gradient intensity, the diffusion coefficient, and the diffusion measurement time.

For a constant linear gradient and diffusion time T1, the echo signal intensity of the n^th^ echo is written as follows:

(2) $$S\;(n\;\mathrm{TE})\;/\;S\;(O)\;=\;\exp[(-n\;\mathrm{TE})/T_{2}]\;\exp[(-\gamma^{2}\;G^{2}\;D.n.\;{\mathrm{TE}}^{3}/\mathrm{T2}]$$

Where γ is the gyromagnetic ratio of the proton

D is the diffusion coefficient

G is the magnitude of the diffusion gradient pulses

Introduction of pulsed gradients instead of a constant gradient, provided a breakthrough for diffusion sensitizing by Stejskal and Tanner in 1965.[6] The pulse sequence used was a spin echo, T2-weighted sequence with two identical magnetic field gradients which dephase and rephase the nuclear spins applied on both sides of the refocusing (RF) pulse. The first gradient is applied between the 90° and 180° RF pulses. Even a microscopic motion after this pulse causes molecules to acquire phase shift; both the 180° pulse and the second gradient pulse rephase the stationary spins (diseased tissues). The phase shift acquired by mobile molecules (healthy tissues) leads to the failure of these molecules to rephase completely, resulting in substantial signal loss, depending on the average diffusion distance and dephasing strength of the gradients. This can be expressed numerically by a product of the diffusion coefficient and the so-called diffusion weighting or degree of diffusion weighting described by the “b” value.

The degree of diffusion-weighting in a diffusion-weighted sequence depends on the strength and the timing of the diffusion gradient.

(3) $$b=\gamma^{2}G^{2}\delta^{2}(\Delta -\delta /3)$$

Where δ = duration of individual diffusion sensitizing lobe, and Δ = time periods between leading edges of the diffusion sensitizing gradient lobes. The resulting signal attenuation can be expressed as:

(4) $$S/S_{o}=\exp(-bD)$$

(5) $$\ln S/S_{o}=-bD$$

S_o_ = Signal without application of diffusion sensitizing lobes

The general equation for the root mean square displacement of water, r, is given by:

(6) $$<r^{2}>=2DT$$

In which T is the spin echo diffusion time and equals to Δ − δ / 3.

Equation (6) assumes a homogenous environment for water diffusion, however, there are other incoherent motions too in tissues. Therefore, only the apparent diffusion coefficient (ADC) and not the true diffusion coefficient can be calculated in clinical DWI, and the signal Intensity (SI) of a DWI is expressed as:

(7) $$S=S_{o}\exp(-b.\mathrm{ADC})$$

Equation (7) shows that SI decreases with increasing b values and increasing apparent diffusion coefficients, so that high b value (1000 s/mm^2^) images are diffusion-weighted and those taken at b = 0 s/mm^2^ are nonDWI images. Diffusion-weighted images result in hyperintensity in lower diffusion (ischemic) areas, compared to healthy brain tissues. However, the signal intensity is weighted by the T2, T1, and the proton density too, the relative contributions depending on the sequence parameters. Therefore, to separate the effect of diffusion, it is necessary to map the apparent diffusion coefficient (ADC). By fitting the signal intensity dependence on the diffusion parameters in DW images; ADC maps are obtained which show lower diffusion as lesser pixel intensity areas. As lower diffusion has a higher signal in the DW image, image contrast in ADC maps is usually the opposite to that in corresponding DW images. Diffusivity of water molecules is not the same in all three dimensions in the brain, and is anisotropic at the voxel scale,[7] because of its characteristic orientation into the myelin sheath, axons, and fiber tracts.[8]

To quantitatively measure diffusion, the apparent diffusion coefficient is calculated pixelwise as the logarithm of the signal intensity such that:

(8) $$\mathrm{ADC}=\frac{\ln\mathrm{SI}(0)/\mathrm{SI}(1)}{b_{1}-b_{0}}$$

Where SI(0) and SI(1) are signal intensities obtained with b = 0 s/mm^2^ and b_1_ = 1000 s/mm^2^, respectively, in this study.

Diffusion-weighted MR imaging to investigate the molecular diffusion of water has been utilized in the assessment of hyperacute ischemic stroke.[9–11] Severe decreases in ADC values within the ischemic core and intermediate ADC values in the ischemic penumbra, indicate that the tissue is at risk of infarction but is potentially salvageable.

### Rationale for the study

The time course of ADC has been studied in stroke patients and reports[10–14] describe the persistence of a reduced ADC for at least 4–8 days after onset. However, the time over which the DWI lesion remained visible in these studies was inferred from the scans of different patients taken at different points of time, rather than repeated scans of the same patient, which makes it difficult to be precise about serial ADC value changes. Furthermore, most studies were retrospective and only few scans were obtained for each patient. A review of the time course of ADC changes in the ischemic brain reveals different values for different laboratories.[9,10,12,13] There is paucity of published data for ADC variations occurring 90 days and later. The purpose of this work therefore was, to study the time course of ADC changes at (1) The hyperacute stage (< 12 h of symptom onset) at about 30 days (subacute stage) and then at 90 days (chronic stage), (2) create a quantitative model for the signal intensity vs time in DW images, and (3) to determine the time for normalization of signal intensity in acute ischemic stroke.

## Materials and Methods

This study was done between 2001 and 2004 on 27 patients attending the Medical Emergency services of our hospital. The patients were selected on a random basis from those at risk for acute cerebral ischemia. Patients whose clinical presentation was suggestive of acute stroke, underwent a noncontrast CT scan of the head immediately, or within three hours of presentation. Those found negative for acute stroke on computed tomography imaging were subjected to diffusion MR imaging, in addition to conventional T_1_ W, T_2_ W, and FLAIR. DW imaging was performed as early as possible, but not later than 12 hours of symptom onset. The onset of ischemia was judged clinically from the appearance of any of the following symptoms viz, aphasia, loss of power in limbs, dysarrythria etc. The time since stroke onset was estimated from the last time the patient was known to be without neurological deficit and was rounded to the nearest hour. DWI and conventional MR imaging was repeated at approximately 30 (subacute stage) and 90 days (chronic stage) in the same patient.

MR studies were performed on a 1.5 T MR system (Magnetom ‘Vision’, Siemens) operating at 63.86 MHz, with gradients of 25 mT/m using phased array coils. .Axial images were acquired (slice thickness = 5 mm and interslice gap = 0.30 mm, 19 images), along the line joining the anterior and posterior commissures. Spin Echo T1 parameters (TR = 665 ms, TE = 14 ms, number of acquisitions = 2, flip angle = 90°) and the corresponding turbo spin echo T2 sequence (TR = 4100 ms, TE = 99 ms, acquisitions = 2; FLAIR sequence (TR = 9000 ms, TE = 110 ms, TI = 2500 ms, acquisitions = 2, flip angle 180°), were acquired in all subjects. The images were acquired in an interleaved manner (with a slice gap of 0.3 mm to reduce cross talk artifacts) with a rectangular FOV of 210 × 240 and a matrix size of 198 × 256. Oversampling was done in the frequency-encoding gradient, but not in the phase-encoding gradient.

A fat-suppressed ,T2-weighted EPI sequence was performed with a TR/TE of 5100/137 ms, a slice thickness of 5 mm; an interslice gap of 0.30 mm, acquisitions = 1; matrix 128 × 128; and FOV of 240 × 240 mm with a gradient overdrive. Twenty images were obtained using Stejskal-Tanner diffusion-encoding gradient pairs in three principal axes (x, y, and z). T2-weighted images were also acquired with constant magnetic field (B_o_) at b = 0 s/mm^2^. CT scans were obtained on a multislice CT scanner (SOMATOM PLUS volume Zoom) with a slice thickness = 5 mm, KVp = 120, and mAs = 400. Seven scans were acquired starting from the base of the skull, resulting in a total of 28 images on a routine head sequence.

ADC Estimate: A small ROI was placed in the centre of the infarct (core) in the diffusion images, and also at six points surrounding the core within the penumbral region. As a control, a second ROI of the same size as the first, was placed in the normal contralateral hemisphere in the same location.

Relative Signal Intensity (DWI): The relative signal intensity of the lesion in the diffusion image (at highest b value = 1000) was defined as:

Relative Signal Intensity (DWI) $({\mathrm{rSI}}_{\mathrm{DWI}})=\frac{{\mathrm{SI}}_{\mathrm{DW}}\;\mathrm{lesion}}{\mathrm{SI}{}_{\mathrm{DW}}\;\mathrm{contralateral}}$

Where SI_DWI_ lesion = the mean Signal Intensity in diffusion images, at b = 1000 s/mm^2^ in the core of the infarct and SI_DWI contralateral_ = signal Intensity in the normal contralateral region on the same image.

Relative Signal Intensity on DWI at b=0 s/ mm^2^ $({\mathrm{rSI}}_{b=0})=\frac{{\mathrm{SI}}_{b=0}\mathrm{lesion}}{\mathrm{SI}{}_{b=0}\mathrm{contralateral}}$

Where S_b=0_ lesion essentially means signal intensity in nondiffusion-weighted images. Each ROI was drawn approximately two pixels inward from the lesion edge excluding the CSF. Where multiple lesions were seen in same hemisphere, each was outlined individually.

ADC values were calculated using the Stejskal-Tanner Equation: $\mathrm{ADC}=\frac{-\ln S_{1}/S_{0}}{b_{1}-b_{0}}$

Where S_0_ and S_1_ signal intensities were obtained with b = 0 s/ mm^2^ and b_1_ = 1000 s/mm^2^.

The relative ADC of the lesion was defined as: $\mathrm{rADC}=\frac{{\mathrm{ADC}}_{\mathrm{lesion}}}{{\mathrm{ADC}}_{\mathrm{control}}}$

**Graph 1:**
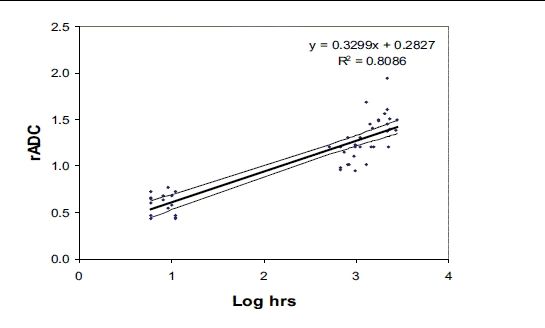
Scatter plot of rADC vs log hours since onset; thick line represents the linear regression that fit the data

Lesion ADC was compared with control ADC values for three time periods: < 12 h; at 30 days (approximately), and at 90 days (approximately), and the rADC values were compared with the logarithmic values of time (hour) from the onset of symptoms. A linear regression model was arrived at for these data and the time at which the regression line intercepted the value of 1 was determined from this graph [Graph 1].

Using the data for all patients, the log values of SI _DWI_ were modeled as a function of the time interval (in log hours) that had elapsed between the time of onset and the time of imaging. A simple regression analysis method was used after transforming the time and SI_DWI_ (Scatter plot) on a logarithmic scale to base 10. To determine the signal intensity in a b = 0 image, the T2 effect was defined as SI_b0_, and the log of SI_b0_ was modeled as a function of time. The time at which psedonormalization occurred was determined. For statistical analysis, paired analysis of the differences was performed with Student's t-test. ADC values of the lesions and control tissues were compared using a two-tailed paired ‘t’ test, with *P* < 0.05 considered to be significant.

## Results

Diagnosis of cerebral ischemia was confirmed in 24 patients in follow-up studies, and repeated at 38 ± 10.7 and 83 ± 18 days. Three patients scanned by DWI within twelve hours of stroke onset, had no evident lesions. The mean time between the onset of stroke and earliest MR was 8.33 h (range: 5–11 h). Nineteen patients had one follow-up study and only sixteen patients came for a second follow-up study. Within twelve hours, the mean ADC value (± SD) in the core of the infarct was 6.32 ± 2.09 × 10^−4^ mm^2^/sec (*P* < 0.001, Table 1).

**Table 1:** Mean ADC values and ratios of ADC ischemic lesions in the core and corresponding contralateral region in acute, subacute, and chronic phases (rADC = ADCI / ADCc)

*Time*	*ADC lesion*	*ADC contralateral*	*rADC*	*P value*
< 12 hours	6.32 ± 2.09	10.46 ± 2.30	0.59 ± 0.11	< 0.001
∼ 30 days	10.35 ± 1.99	9.15 ± 0.36	1.12 ± 0.13	0.002
∼ 90 days	13.6 ± 2.22	9.50 ± 1.11	1.48 ± 0.18	0.001

Evolution of ADC characteristics at the core of infarcts; the ADCI (ADC lesion) and ADCc (ADC contralateral region) were compared using two tailed paired t test

The mean ADC of the ischemic lesion was 41% lower than the mean ADC compared to the contralateral hemisphere (ADC contralateral). For lesions in the late subacute stage that had been scanned at 38 ± 10.7 days, the mean ADC was 10.35 ± 1.99 × 10^−4^ mm^2^/sec, compared to the mean ADC values of 9.15 ± 0.36 × 10^−4^ mm^2^ sec (*P* = 0.002) within the control region, or 12% above the normal value. Chronic infarcts scanned at 83 ± 18 days had a mean ADC value of 13.6 ± 2.22 compared to the mean ADC value of 9.50 ± 1.11 within contralateral region or 48% above the normal value (*P* < 0.001). In the acute phase, the mean rADC (± SD) value at the core of the region was 0.59 ± 0.11 whereas the mean rADC (± SD) in the penumbral region was 0.92 ± 0.10 (*P* < 0.001). The subacute stage (at ∼30^th^ day) had an rADC value of 1.12 ± 0.13 whereas the chronic phase (at ∼ 90^th^ day) had an rADC value of 1.48 ± 0.18.

The data for rADC *vs* time in log hours since onset are plotted in Graph 1. Linear regression analysis of rADC *vs* log hours shows that pseudonormalization occurred at 6.61 days (*P* < 0.001 with a slope of 0.32 and R^2^ = 0.81).

A representative case of an ischemic infarction is shown in Figures 1–5 and demonstrates the evolution of a right MCA infarction nine hours after onset. The lesion was hyperintense in diffusion-weighted images and hypointense in the corresponding ADC map. On day 35, the lesion increased in size in the first follow-up study, and was still hyperintense in DW images [Figures 6–10]. On day 90, the ADC of the infarct had pseudonormalized and the lesion became isointense compared to normal tissues [Figures 11–15].

Table 2: Time course of the T2 effect (rSI _b=0_ indicates relative T_2_-weighted signal intensity (b=0 s/mm^2^ ) at the ischemic core

**Figure 1:**
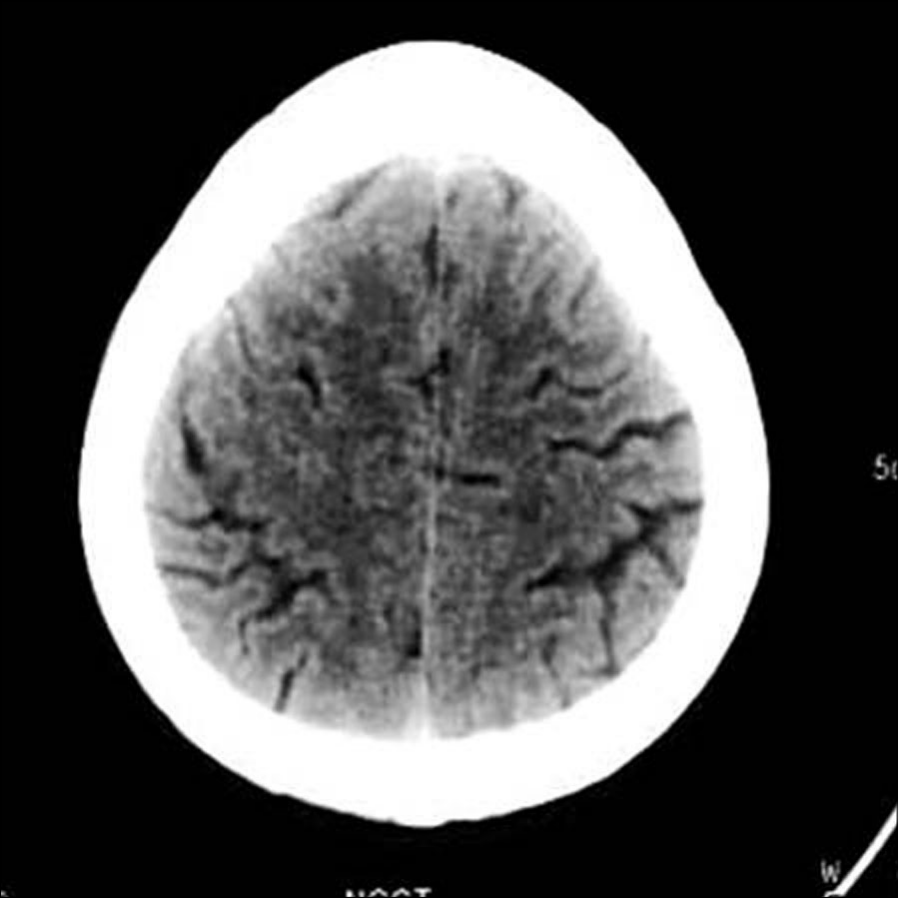
Axial CT scan of a patient not showing any focal lesion (study case Images taken 9 h after ischemic stroke)

**Figure 2:**
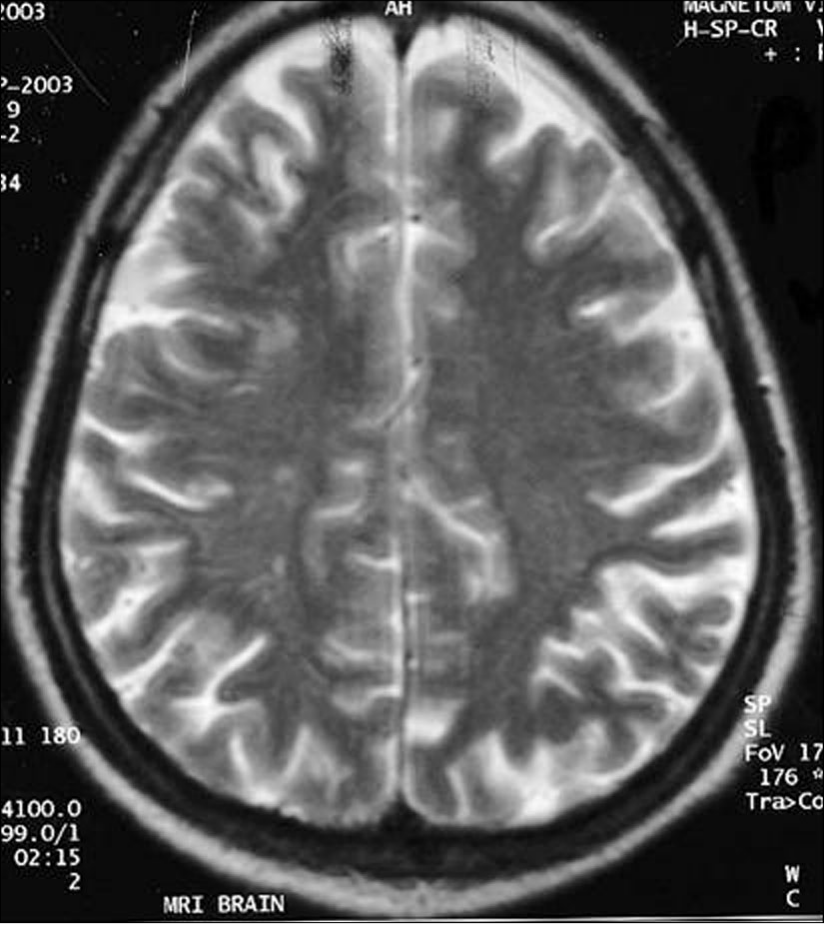
Discrete hyperintense lesion is seen in right frontal white matter and faint hyperintensity seen in the right frontoparietal gray white matter junction in t2w image (study case images taken 9 h after ischemic stroke)

**Figure 3:**
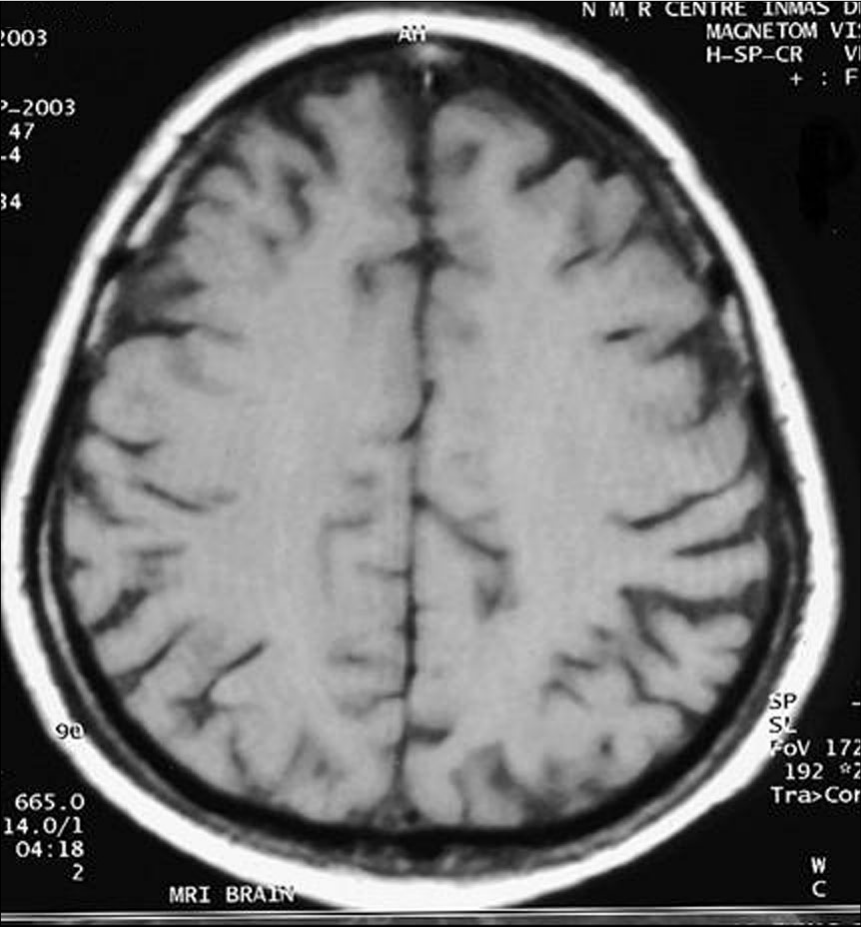
Same lesion was isointense in t1w image (study case images taken 9 h after ischemic stroke)

**Figure 4:**
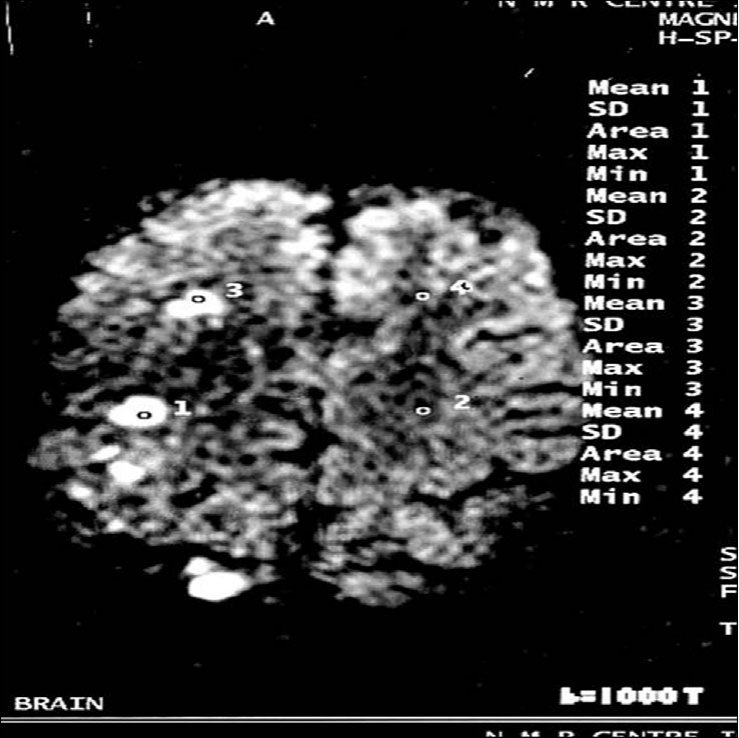
Diffusion-weighted image b = 1000 showing discrete hyperintense foci seen in right frontal white matter and in right frontoparietal region (study case images taken 9 h after ischemic stroke)

**Figure 5:**
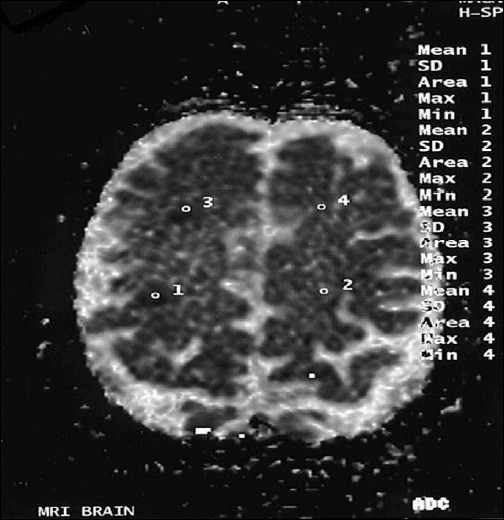
These lesions appear hypointense in ADC map (study case images taken 9 h after ischemic stroke)

**Figure 6:**
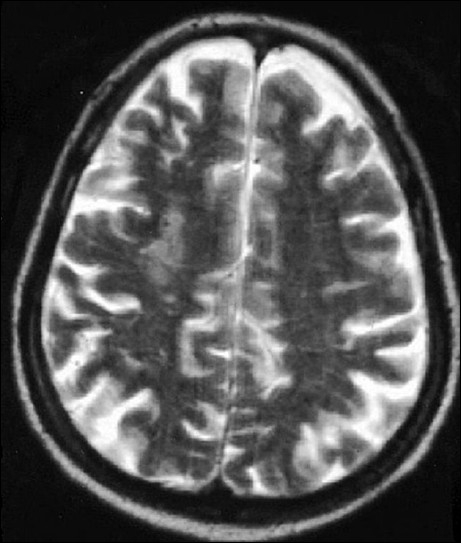
Right frontal lesion is more well defined and hyperintense in T2W image (first follow-up study of same patient done 35 days after acute onset)

**Figure 7:**
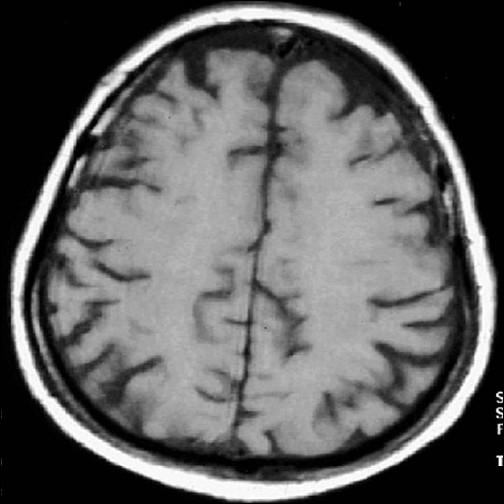
The lesion appears mildly hypointense in T1W image (first follow-up study of same patient done 35 days after acute onset)

**Figure 8:**
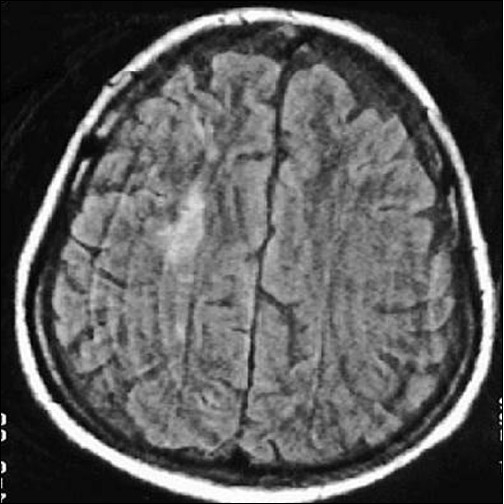
FLAIR sequence shows more intense lesion as compared to initial study (first follow-up study of same patient done 35 days after acute onset)

**Figure 9:**
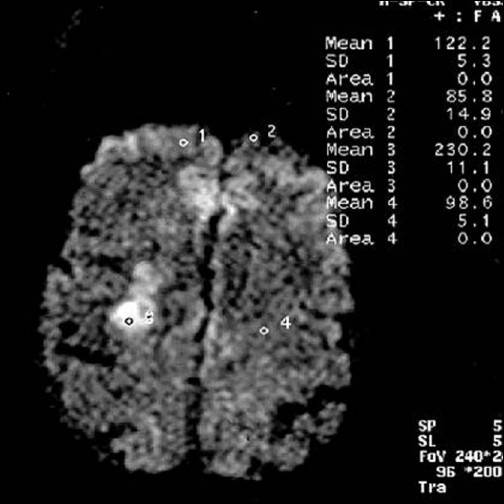
Right frontal lesion appears less bright and right frontoparietal lesion brighter with increased in volume (first follow-up study of same patient done 35 days after acute onset)

**Figure 10:**
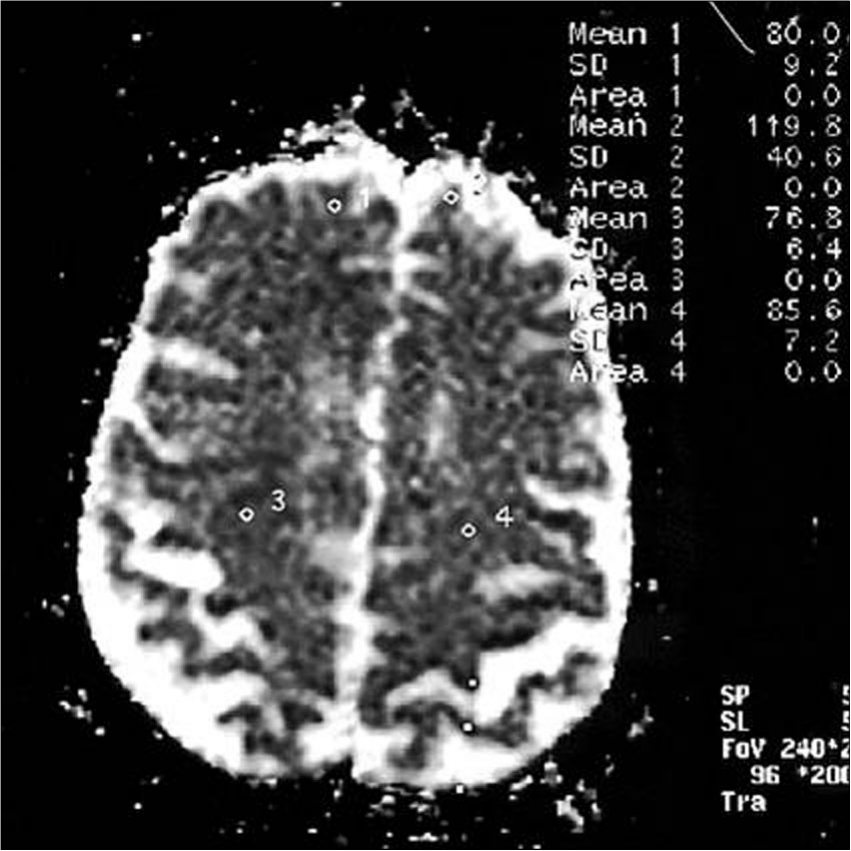
Lesions now appear brighter in ADC map as compared to initial study (first follow-up study of same patient done 35 days after acute onset)

**Figure 11:**
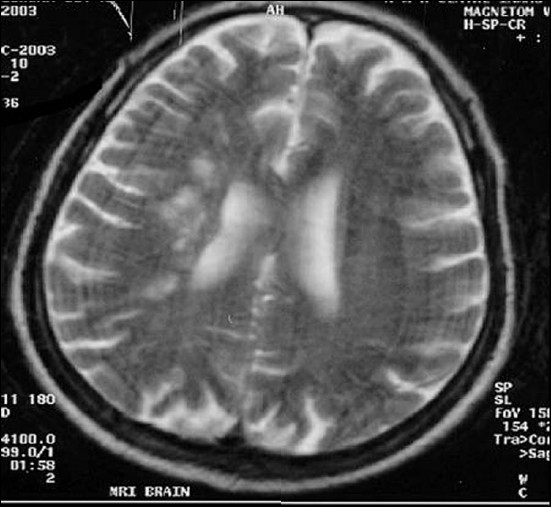
Second follow-up study shows progression of same lesion in T2W mage (second follow-up study of the same patient done 90 days after acute onset)

**Figure 12:**
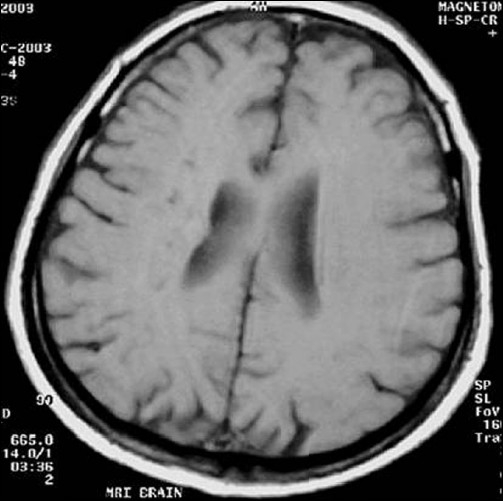
Second follow-up study shows progression of same lesion in T1W mage (second follow-up study of the same patient done 90 days after acute onset)

**Figure 13:**
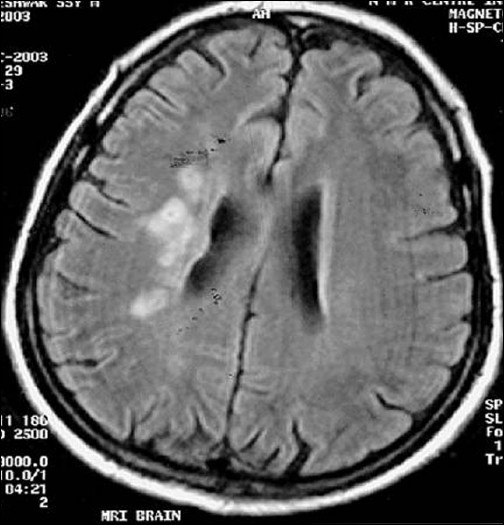
Second follow-up study shows progression of the same lesion on FLAIR image (second follow-up study of the same patient done 90 days after acute onset)

**Figure 14:**
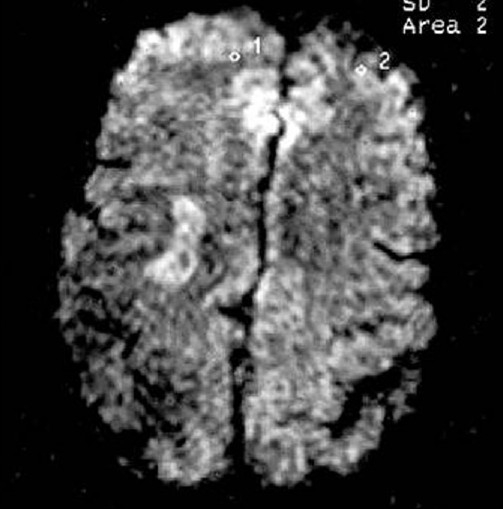
Chronic infarcts (diffusion-weighted images) suggest improved molecular diffusion of water, apparent as decreased hyperintensity in DWI at b = 1000 (second follow-up study of the same patient done 90 days after acute onset)

**Figure 15:**
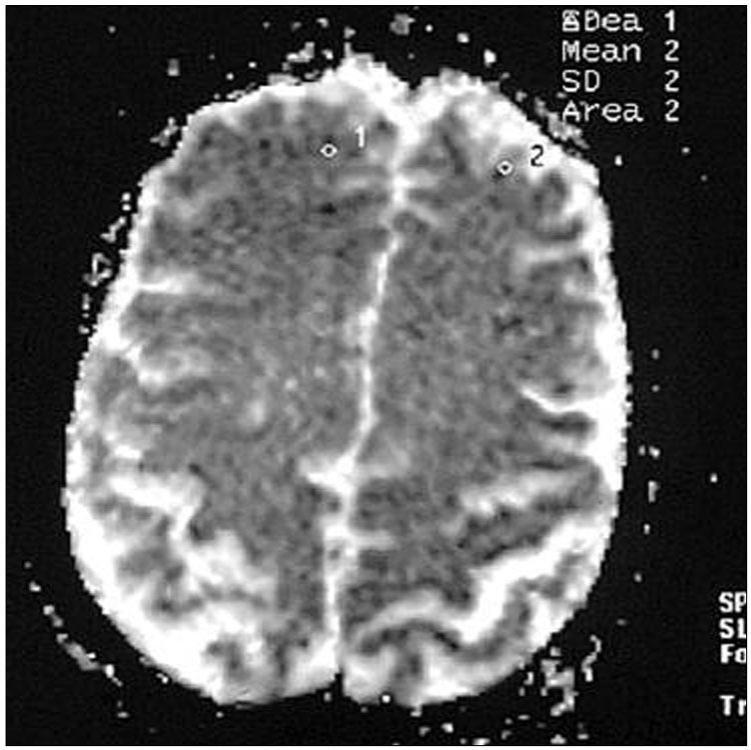
ADC map of the same patient in the chronic stage 90 days after infarct (lesion appear more hyperintense)

**Table 2:** Time course of the T2 effect (rSI b=0 indicates relative T_2_-weighted signal intensity (b=0 s/mm^2^) at the ischemic core

*Time*	*rSI b=0*
< 12 hours	1.63 ± 0.19
∼ 30 days	2.01 ± 0.13
∼ 90 days	2.28 ± 0.24

As shown in Table 2, signal Intensity (rSI b=0) within twelve hours of the infarct core was 1.63 ± 0.19. Thereafter, it significantly increased from 1.63 ± 0.20 in the acute stage to 2.28 ± 0.24 in the chronic stage (∼ 90 days) (*P* < 0.001). The mean SI b=0 was 259±64in the control region [not shown in Table 2] at approximately day 30; the ratio of signal intensity was 2.01 ± 0.13. The signal intensity at b=0 (SIb=0) was significantly higher than control values for all time periods. However, the hyperintensity was so subtle in the baseline MR study that the lesion could not be identified by visual inspection alone, and the region of interest (ROI) from the DWI at b = 1000 s/mm^2^ image was transferred to the diffusion images (at b = 0) for signal intensity measurements.

**Graph 2:**
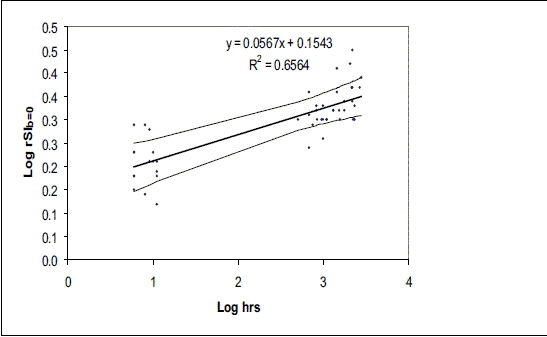
Scatterplot of the log of SI b=0 vs log hours since symptom onset; the thick line represents the line of best fit and thin line lines represent 95% CI

Graph 2 is a linear regression graph of the ratio of signal intensity at b = 0 (rSI _b=0_ s/mm^2^ ) *vs* the time interval expressed logarithmically between onset and imaging. The fitted curve derived from our data revealed the value of rSI _b=0_ at the time of pseudonormalization to be 1.91.

In this study, rSI_DWI_ decreased from 2.54 ± 0.46 in the acute stage to 1.54 ± 0.22 in the chronic stage [Table 3]. The patients imaged at 38 ± 10.7 days had a rSI_DWI_ value of 1.84 ± 0.34. The mean signal intensity in DWI at b = 1000 s/mm^2^ (SI_DWI_) was significantly higher than the control values throughout all periods. The lowest rSI_DWI_ was 1.01 and was seen in the lesion imaged 93 days after symptom onset. Fourteen of the sixteen patients with an infarct older than 30 days had decreased SI_DWI._.

**Table 3:** Signal Intensity values of rSIDWI of ischemic lesions at different time intervals

*Time*	*rSIDWI*
< 12 hours	2.54 ± 0.41
∼ 30 days	1.84 ± 0.34
∼ 90 days	1.54 ± 0.22

Evolution of time course of infarct signal intensity in DW image; (rSIDWI, represents relative diffusion-weighted signal intensity (b = 1000 s/mm^2^)

**Graph 3:**
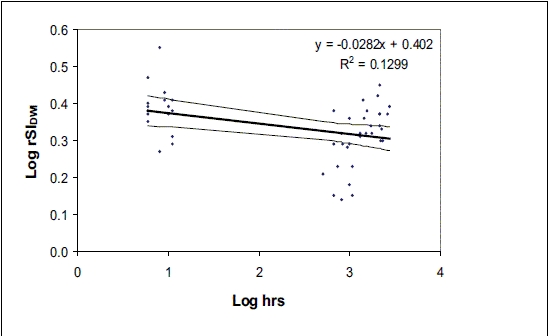
Scatter plot depicting the log of the ratio of lesion signal intensity in image (b = 1000 s/mm2) to the lesion signal intensity in the image with b = 0

The data shown in Graph 3 where log SI_DWI_ values were plotted with time in log hours shows that the value of rSI_DW_ was found to be 2.14 at the time of pseudonomalization.

## Discussion

The present study showed that the ratio of signal intensity at b = 0(rSI_b0_ ) of an ischemic lesion progressively increased from the acute stage (1.63 ± 0.20) to (2.28 ± 0.24) in the chronic stage (approx. 90 days). The signal intensity (rSI_b0_) was significantly higher than the control values at all time periods. Our results are in agreement with the findings of Lansberg *et al.*, [10] who reported signal intensity SI_T2W_ of 1.51 ± 0.21 in the acute stage and 1.98 ± 0.32 in lesions older than 14 days. The slight difference in the values of signal intensities may be due to the use of different techniques and a different patient population. The early rise in SI_b0_ makes it possible to differentiate acute lesions from chronic lesions. Lesions with an rSI_b0_ < 1.65 were considered to be acute and rSI_b0_ > 1.65 were considered to be chronic from the fitted curve derived from our data [Graph 2]. Psedonormalization corresponded to an rSI_b0_ value of 1.91. This result is comparable with result of Lansberg *et al.* of 1.88 ± 0.25 at around 5–7 days after stroke onset.

In the present study, the relative signal intensity in DWI progressively decreased from 2.54 ± 0.46 in the acute stage to 1.54 ± 0.22 in the chronic stage and the mean SI_DWI_ was significantly higher than the control value throughout this time period. The increased relative signal intensity that was seen in the diffusion-weighted images in the acute stage was indicative of the more restricted diffusion of the water within the intracellular environment in ischemic tissues. Similar to our findings, Lansberg *et al.*[10] also reported that SI_DWI_ decreased from 1.90 ± 0.34 in the acute stage to 1.50 ± 0.16 in infarcts older than 14 days. In the present study, lesions remained hyperintense at the time of the first follow-up. This pattern was most likely the result of SI_DWI_ being influenced by two factors, mainly the water diffusibility and the intrinsic T2 properties of the tissue being examined.

Decreased rADC in the core of infarct, seen by us in the acute stage, matches with those previously published by Schlaug *et al.*[13] The rADC decreased significantly in acute lesions and tended to progressively increase thereafter. Increased rADC values in subacute (∼30 day) and chronic phases (90 days) also match the published results.[14] Lansberg *et al.*[14] reported rADC values of 1.37 ± 0.31 in 27 lesions imaged 14 days after onset. Values seen by us were found to be 1.12 ± 0.13 and 1.48 ± 0.18 in patients imaged at 38 ± 0.33 and 88 ± 10.66 days since onset. According to our simple linear model, pseudonormalization of infarct ADC values which occurred on 6.61 days after onset, matches with reports of previous studies, where such pseudonormalization typically occurred between the seventh and 11^th^ day after onset.[13]

## Conclusions

This study documents the time course of signal intensity SI_T2w_, SI_DW_ , and ADC in diffusion-weighted imaging. Apparent diffusion might assist the clinician in selecting patients with potentially ischemic brain regions within the penumbra. The mean rADC found in this study was 41% below that of normal tissue in the core of the infarct. The infarct core has an rADC < 0.75. Regions of the penumbra with intermediate rADC values are at the greatest risk of infarction. We believe that data from the present study concerning the normalization of infarct signal intensity can also be applicable in other patient populations. The area of the penumbra of the infarct is potentially salvageable during a window period of up to six hours.

Diffusion-weighted MR imaging as shown in the study reveals the altered ADC values during this time period and if combined with MR perfusion imaging (which shows no loss of perfusion in the area surroundings core of infarct), it can identify potentially salvageable brain tissues at an early stage. This study can also help interventional radiologists in selecting cases for such therapies for stroke management.
